# Fear of infection or fear of protection – driving factors of COVID-19 vaccine uptake in Bulgaria

**DOI:** 10.1093/eurpub/ckac130.230

**Published:** 2022-10-25

**Authors:** M Rohova, R Pancheva, NL Mihaylov, N Radeva, E Ivanova, R Chamova, Ts Paunov, M Kolarova, S Hadzhieva

**Affiliations:** 1 Department of Economics and Health Care Management, Faculty of Public Health, Medical University, Varna, Bulgaria; 2 Department of Hygiene and Epidemiology, Faculty of Public Health, Medical University, Varna, Bulgaria; 3 Department of Disaster Medicine and Maritime Medicine, Faculty of Public Health, Medical University, Varna, Bulgaria

## Abstract

**Background:**

Bulgaria faced significant COVID-19 morbidity and mortality rates, but many people underestimated the risk of transmission and severity of infection. Rising vaccine-related fear and misinformation exacerbated existing hesitancy and mass vaccination remained a challenge. This study aims to investigate the factors influencing COVID-19 vaccination uptake in Bulgaria.

**Methods:**

A cross-sectional study was conducted in April 2022 in a sample of 1,200 respondents. Data were collected via an online self-administered questionnaire, measuring perceived risk of COVID-19, vaccine attitudes, trust in health system, and sociodemographics. Results were analyzed using bivariate and multivariate statistical methods.

**Results:**

Bivariate analyses showed that the majority of vaccinated respondents (81.9%) expressed concern about infection, compared to 47.1% of non-vaccinated. Significant differences were related to perceived risk of COVID-19 vaccine: 61.0% of vaccinated assessed risk as small versus 7.4% of refusers. Non-vaccinated participants demonstrated distrust in vaccine benefits and lack of trust in health system, science and pharmaceutical companies. The multivariable regression revealed associations between age, income, vaccine perception, and vaccination uptake. The 45-54 and 55-64 age groups were less likely to refuse vaccination compared to the youngest age group (OR = 0.34, p = 0.009 and OR = 0.38, p = 0.036). Odds of refusing the COVID-19 vaccine decreased as income increased (OR = 0.33, p = 0.036). A high perceived risk of adverse effects increased the odds of vaccine refusal by 7.02 (p<.001).

**Conclusions:**

The lack of confidence in the vaccine safety and effectiveness, coupled with an underestimation of the coronavirus disease, formed a critical barrier to the vaccine uptake. The misinformation fueled vaccination fear. Public health campaigns should address vaccine-related concerns and promote vaccination adherence in more consistent manner targeting also the spread of fake news.

**Key messages:**

- The lack of confidence became a crucial barrier for vaccination coverage in Bulgaria.
- Understanding driving factors for vaccine uptake is of utmost importance to design effective vaccination programs.

